# Randomized clinical trial of surgery versus conservative therapy for carpal tunnel syndrome [ISRCTN84286481]

**DOI:** 10.1186/1471-2474-6-2

**Published:** 2005-01-18

**Authors:** Brook I Martin, Linda M Levenson, William Hollingworth, Michel Kliot, Patrick J Heagerty, Judith A Turner, Jeffrey G Jarvik

**Affiliations:** 1 Department of Medicine, Division of General Internal Medicine, Multidisciplinary Clinical Research Center, Box 359736, 325 Ninth Ave. Seattle, Washington, 98104, USA; 2 Department Psychiatry and Behavioral Sciences, University of Washington, Box 356560, 1959 NE Pacific Street, Seattle, Washington, 98195 USA; 3 Department of Radiology, University of Washington, Box 357115, 1959 NE Pacific Street, Seattle, Washington, 98195, USA; 4 Department Neurological Surgery, Harborview Medical Center, Box 359766, 325 Ninth Ave., Seattle, WA 98104, USA; 5 Department of Biostatistics, University of Washington, Box 357232, 1959 NE Pacific Street, Seattle, Washington, 98195, USA

## Abstract

**Background:**

Conservative treatment remains the standard of care for treating mild to moderate carpal tunnel syndrome despite a small number of well-controlled studies and limited objective evidence to support current treatment options. There is an increasing interest in the usefulness of wrist magnetic resonance imaging could play in predicting who will benefit for various treatments.

**Method and design:**

Two hundred patients with mild to moderate symptoms will be recruited over 3 1/2 years from neurological surgery, primary care, electrodiagnostic clinics. We will exclude patients with clinical or electrodiagnostic evidence of denervation or thenar muscle atrophy.

We will randomly assign patients to either a well-defined conservative care protocol or surgery. The conservative care treatment will include visits with a hand therapist, exercises, a self-care booklet, work modification/ activity restriction, B6 therapy, ultrasound and possible steroid injections. The surgical care would be left up to the surgeon (endoscopic vs. open) with usual and customary follow-up. All patients will receive a wrist MRI at baseline.

Patients will be contacted at 3, 6, 9 and 12 months after randomization to complete the Carpal Tunnel Syndrome Assessment Questionnaire (CTSAQ). In addition, we will compare disability (activity and work days lost) and general well being as measured by the SF-36 version II. We will control for demographics and use psychological measures (SCL-90 somatization and depression scales) as well as EDS and MRI predictors of outcomes.

**Discussion:**

We have designed a randomized controlled trial which will assess the effectiveness of surgery for patients with mild to moderate carpal tunnel syndrome. An important secondary goal is to study the ability of MRI to predict patient outcomes.

## Background

Carpal tunnel syndrome (CTS) is the most common peripheral nerve entrapment syndrome, with an annual incidence of 50–150 cases/100,000. It is an important cause of workplace morbidity [1] with approximately 30,000 cases of CTS resulting in days lost from work in 1996 (BLS US Department of Labor 1998; [2]). Typical symptoms include paresathesia, pain, and weakness in a median motor nerve distribution, which are often worse at night [3,4]. Although frequently idiopathic, CTS may be associated with diabetes, thyroid disorders, pregnancy, renal dialysis, and other conditions [5].

A Cochrane literature review [6] for randomized trials that compared surgical to non-surgical treatment of CTS included only one article [7]. This study demonstrated significant clinical improvement in electromyography and symptoms reported at 1 year for surgical release over splinting with a cohort of 22 women. More recently, Gerritsen et al, published a second randomized study of surgical release versus splinting in 176 patients with moderate carpal tunnel syndrome, defined by clinical and electrophysiological testing [8,9]. Surgical patients had greater improvement in the number of nights waking up due to symptoms, and severity of symptoms, as well as on a general improvement scale. However, the evidence is less clear for patients with a shorter duration of symptoms or the use of conservative therapies other than splinting, such as physical therapy and ultrasound [9,10].

Although there is generally a lack of rigorous scientific support for non-surgical treatments for CTS [2], there is limited evidence of benefit for certain interventions. Common conservative treatments for CTS include wrist splints, hand therapy, non-steroidal anti-inflammatory drugs (NSAIDs), and corticosteroid injection into the carpal tunnel [11-26]. Garfinkel [15] reported that yoga hand exercises resulted in improved Phalen sign compared with splints. Rozmaryn [14] reported that nerve and tendon gliding exercises coupled with traditional conservative care may reduce the need for surgery. Little evidence exists to support the use of NSAIDs for treating CTS. Celiker et al [16] found that corticosteroid injection into the carpal tunnel was superior to NSAID therapy and Davis and colleagues [12,13] reported that ibuprofen combined with nocturnal splints did not improve outcomes more than chiropractic manipulation [15,17-19]. Local injection of corticosteroid into the carpal tunnel improves short-term clinical outcomes, as compared with oral corticosteroids, intramuscular corticosteroid injections, NSAIDS, or splints alone. [16,20-23]. Herskovitz and colleagues [24] demonstrated a short term improvement in global symptom scores for CTS with oral corticosteroid compared to placebo, but Chang could not find a dose response [25,26]. While these finding suggest some potential therapeutic benefits, none of these therapies either alone or in combination have been rigorously compared to surgery.

Although not commonly used, Ebenbichler [27] found that focused ultrasound significantly improved symptom and electrophysiological outcomes compared with sham ultrasound. Vitamin B-6 has also been suggested as a treatment for CTS, [28-32] but two studies [33] failed to demonstrate improvement in outcome.

Diagnostic criteria for grading CTS severity as "mild" and "moderate" are not well established. Electrodiagnostic studies (EDS) do not correlate well with clinical severity and have not been shown to accurately predict outcomes for patients with mild to moderate CTS [34-42].

We designed a randomized clinical trial to compare surgical release to non-surgical treatment for patients with mild to moderate CTS. We will examine the association between outcome, as measured by symptoms and functional status, and baseline variables such as symptoms, function, occupational risk factors, EDS measures, demographics, signs and symptoms, and prior treatments. Our primary endpoint is at 12 months.

In addition, our study will evaluate the ability of high resolution magnetic resonance imaging (MRI) of the wrist to predict who will benefit from surgical release. MRI has the potential to offer new insight into the diagnosis and management of patients with hand and wrist neurological symptoms. Unlike electrodiagnostic studies, MRI directly visualizes the median nerve and can detect abnormalities of both configuration (nerve compression) as well as signal (indicating intraneural edema and demyelination) [43-46]. Either or both of these findings have the potential to be better predictors of patient outcomes than electrodiagnostic studies.

Finally, we will gather utilization data for each arm of the study to test the hypothesis that the incremental cost-effectiveness ratio of surgery falls below conventional cost-effectiveness thresholds.

## Methods/design

We have designed a multi-center, randomized trial comparing surgical release to a multi-component, non-surgical therapy. The study protocol was approved by the University of Washington Human Subjects Division and all participants provide written informed consent.

The two major goals of this study are to determine: 1) if surgery compared with conservative therapy benefits patients with mild or moderate carpal tunnel syndrome, and, 2) if high resolution magnetic resonance imaging (MRI) of the median nerve can identify patients for whom early surgery might be more efficacious than conservative therapy.

Patients with mild to moderate CTS are recruited from six participating sites in the Puget Sound region of Washington State: the University of Washington (UW) affiliated practice sites (University of Washington Medical Center, UW Physicians Network, Harborview Medical Center, Puget Sound VA Health Care System), Virginia Mason Medical Center (Seattle), the Seattle Hand Surgery (affiliated with Swedish Hospital in Seattle), Proliance Surgeons (affiliated with Overlake and Evergreen Hospitals), and Management Services Organization of Washington in Tacoma. We recruit patients within the primary care clinics as well as the specialty referral clinics that treat patients with carpal tunnel syndrome. These clinics include neurological surgery, neurology, orthopedic surgery, and physical medicine and rehabilitation. Additionally, we identify potential subjects in the electrophysiology laboratories at each of our participating sites.

Prior to study participation, patients are required to have a physician confirmation of suspected CTS and to obtain an EDS with or without an electromyeogram (EMG). We define mild to moderate carpal tunnel syndrome based on electrodiagnostic studies (EDS) and clinical findings. Specifically, patients are eligible for the study if EDS demonstrates any one of the following: (1) wrist median motor nerve conduction latency greater than or equal to > = 4.4 milliseconds (ms), (2) a 10 cm (thumb to wrist) median to radial sensory nerve ratio difference of > 0.5 ms, (3) an 8 cm mid-palm median to ulnar sensory nerve difference > 0.3 ms, (4) a 14 cm (digit four to wrist) median to ulnar difference of > 0.4 ms, or (5) a combined sensory index [47,48] ≥ 1.0 ms. Patients with normal EDS findings could still qualify for the study if they reported hand symptoms at night that awakened them, a positive flick test, and a "classic", "probable" or "possible" evaluation of a hand diagram. [49,50]

Other study inclusion criteria are: age 18 years or older, no previous hand or wrist surgery on the study hand, no previous carpal tunnel release on the contralateral hand in the previous 6 months, symptoms in at least two digits in a median motor nerve pattern, able and willing to answer research questionnaires in English, and classification using the hand diagrams as at least "possible CTS"[49,50]. Exclusion criteria are prior CTS release on the involved hand; diffuse peripheral neuropathy, any known mass, tumor or deformity; any history of severe trauma to the wrist (such as fracture); a deformity of the study hand; and pregnant or lactating (table 1).

**Table 1:** Eligibility Criteria

**Inclusion**		**Exclusion**
Symptoms in at least two digits on one hand (to include thumb, index, middle, or ring finger.)		Wrist or hand surgery within last 6 months
Hand/ wrist symptoms >1 week		Previous CTS release on study hand
Expect to stay in area for 1 year.		Moderate to severe arthritis involving hand or wrist
Willing and able to complete phone interviews		Known tumor, mass, or deformity in the hand or wrist
Over age 18		History of severe trauma to the wrist.
Able to complete questionnaires in English		Pregnant or lactating
Any one of these EDS findings	Motor: Median motor latency (wrist) > = 4.4 ms	Median Motor amplitude <= 3.8 mV
	Sensory: median-radial (10 cm thumb to wrist) difference >0.5 ms	EMG (if done) evidence of denervation
	Sensory: Midpalm median-ulnar (8 cm) difference >0.3 ms	Evidence of diffuse peripheral neuropathy.
	Sensory: Median-ulnar (14 cm digit IV to wrist) difference >0.4 ms	Thenar atrophy
	Sensory: Combined Sensory index > = 1.0 ms	
	(With normal EDS) Night pain that wakes patient AND Positive flick test	
Classic, probable, or possible hand pain diagram.		
Two consecutive weeks of standard (non-surgical) treatment for CTS, including a trial of wrist splints.		
Lack of improvement with conservative treatment documented by at least one of the following:	Improvement less than 0.75 in the CTSAQ functional?	
	Unable to achieve "satisfactory" level of work	
	Patient defined symptoms as being "same" or "worse" over the last two weeks.	
Willing to schedule surgery within one week of randomization.		

Patients with evidence of severe CTS on EDS, EMG, or clinical findings are excluded from the study. Severe CTS is defined as a median motor amplitude of ≤ 3.8 mV, EMG evidence of denervation, or thenar atrophy.

Study participants are required to have not improved after a minimum of two weeks of standard non-surgical treatment (typically, wrist splints and NSAIDs) and be willing to schedule surgery within one week if randomized to the surgical arm of the trial. Lack of improvement with conservative care is defined by any of the following: (1) < 0.75 point improvement [51] on the Carpal Tunnel Syndrome Assessment Questionnaire (see primary outcome measures), (2) self-reported inability to achieve a "satisfactory" level of work due to hand or wrist problems, or (3) self-report of symptoms as "same" or "worse" since they started conservative therapy.

For patients with bilateral CTS, we designate a "study hand" based on the following priorities: 1) most severe according to patient reporting, 2) most severe based on electrodiagnostic reports, and 3) the dominant hand.

We use a 50/50 computer-generated block randomization, stratified by enrollment site. The block size is randomly varied between 4 and 12 to reduce the potential for clinicians or research staff to predict treatment allocation. The treatment assignment is centrally administered and concealed in consecutively numbered opaque envelopes.

Patients are randomized to receive either a surgical release of the median nerve or a package of multiple, non-surgical treatments tailored for individual patients. For those randomized to surgery, we attempt to schedule the surgery within two weeks of allocation or as soon as possible. Surgery is performed by a board-certified neurological surgeon or orthopedic surgeon. Either open or endoscopic surgery can be performed, depending on the surgeon's preference. Surgical patients receive clinical follow-up just as they would if not in the study. Typically, this includes a follow-up visit within one week for wound and suture management and several post-operative follow-up visits with a hand therapist to perform median nerve and tendon gliding exercises.

We created expert focus groups and reviewed the relevant body of literature to develop the non-surgical treatment arm of study. Our focus group included experts in orthopedics, neurosurgery, physical medicine and rehabilitation, hand therapy, biostatistics, health services, behavioral science, and health economics.

The non-surgical treatment is directed by a hand, occupational, or physical therapist and includes a minimum of three visits, separated by 6 weeks each. At the first visit, each patient receives an educational booklet, a prescription for NSAIDs (if they have not previously tried them), and specific exercises for their hand. The educational booklet details the hand exercises, describes the causes of carpal tunnel syndrome, and lists resources for obtaining additional information. Work and activity modifications are prescribed at the discretion of the hand therapist and additional hand therapy visits are prescribed as needed. Patients allocated to the non-surgical arm who have already undergone extensive physical therapy can opt to get ultrasound treatment immediately (see below).

Patients in the non-surgical arm return six weeks after the randomization for a study visit. If a patient reports improvement, no changes to their therapy are made. For patients who do not improve, we offer oral vitamin B-6 supplements (100 mg per day) and ultrasound in addition to the existing therapy regimen. The ultrasound regimen used in this study consists of up to 12 sessions per week (for 6 weeks) of focused ultrasound at 1 Mhz, 1.0 W/sqr cm^2^, in pulsed mode 1:4, and 15-minutes each. Three months after randomization, patients return for the final non-surgical evaluation. If improved, they are instructed in a self-care maintenance program and advised to return to their provider if their symptoms worsen. If symptoms are not improved, patients are referred to a study surgeon for evaluation for crossover to receive surgery or corticosteroid injection.

Outcomes measures are collected for patients in person at baseline and via telephone interviews at 3, 6, 9, and 12 months after randomization. Although it is impossible to blind providers and patients to the treatment the assignment, the telephone interviewer is blinded to study participants' treatment assignment.

The functional status scale of the Carpal Tunnel Syndrome Assessment Questionnaire (CTSAQ) is the primary outcome measure. The CTSAQ is a self-report CTS functional status and symptom severity questionnaire with established validity, reliability and responsiveness [52,53]. The functional status scale assesses ability to perform eight common tasks involving the hands. The symptom severity scale consists of eleven items assessing symptoms of pain, numbness, and weakness at night and during the day. Each question is answered on a scale of 1 to 5 and the scales are scored by taking the mean of the responses, with a higher score indicating greater severity.

The SF-36 Health Survey version 2 (QualityMetics Inc., Ware) has been used to assess general health status in samples of patients with a variety of diseases, including CTS [54]. It consists of eight domains (general health, physical functioning, role limitations due to physical problems, role limitations due to emotional problems, bodily pain, social function, mental health, and vitality) scored on a scale of 0 (worst health) to 100 (ideal health). We will compare the two groups on each scale as well as the physical and mental summary scores. The generic nature of the instrument allows comparison across health conditions.

Study participants also complete the Symptom Check List SCL-90 12-item Somatization and 13-item Depression scales [55-57]. Participants respond to each question using a 5-point scale ranging from "not at all" to "extremely". Higher scores indicate greater somatization/depressive symptom severity.

The 13-item Pain Catastrophizing Scale is used as both a predictor and a secondary outcome. A substantial volume of research had consistently found substantial associations between pain-related catastrophizing and pain-related disability [58-62]. We are interested in learning whether pain-related catastrophizing is a risk factor for poor outcomes in patients with CTS.

In addition to the outcome measures, we obtain information on several other variables at baseline. This includes information on demographics, occupational risk factors, medical co-morbidity, hand pain history, work status, and litigation/compensation issues. The first three items of the Alcohol Use Disorders Identification Test (AUDIT) are administered to assess the frequency of alcohol use, amount of alcohol consumption, and binge drinking [63-65].

We are also collecting information on the costs of care following enrollment. We aim to estimate the cost to health purchasers and society based on billing and medical records, and a detailed resource use questionnaire (RUQ) at 3, 6, 9, and 12 months post-enrollment.

We attempted to either measure or abstract from the medical records information that is generally included in the hand physical examination at the time of enrollment. The hand physical included measurements of patient height, weight, dominant hand, 2-point discrimination (measured at digits #2 and #5), wrist width and thickness (for MRI correlation), 2-point pinch strength (average of three efforts), Semmes-Weinstein monofilament test (from 1.65 to 6.65), Tinel sign, Phalen sign (held greater than 1 minute), and flick test. Not every site routinely completed all sensibility and/or strength tests.

Mackinnon-Dellon disk-criminators™ are used to test the static two-point discrimination at the second and fifth digits as a measure of sensibility to correlate to the MRI findings. We tested each digit a minimum of three times and until the tester was confident that a clear endpoint was reached. The test was performed on both hands even in patients with unilateral disease. The disk prongs are held perpendicular to the long axis of the finger. The prongs are placed upon the skin only with sufficient pressure for a patient to determine that he is being stimulated. The three-point pinch strength is tested bilaterally, with the non-study hand being first. We recorded the mean of three serial efforts of the key pinch measurement (thumb pad to lateral aspect of middle phalanx of the index finger) using a B&L Engineered (B&L Engineering 3002 Dow Ave, Suite 416, Tuscin CA 92780) 30 lbs. pinch gauge calibrated to +/- 1% accuracy.

Tinel sign is positive if tapping over the median nerve at the distal wrist crease for approximately 10 seconds reproduces the pain, numbness and or tingling in the patients hand or wrist. Phalen sign is positive if while holding both wrists flexed at 90 degrees with the dorsum of the hands in opposition to each other for a minimum of 1 minute produces dysesthesias and pain. A flick test is positive if in their response to being asked "What do you actually do with your hand(s) when your symptoms are at their worst?" a patients gestures by flicking the wrist(s) [66-74].

All subjects who enter the study undergo wrist MRI except for subjects who have MRI contraindications (e.g., metallic hardware within their body), are claustrophobic, who exceed the weight limit of the MRI table, who have scheduling difficulties, or refuse the MRI. We use phased-array surface coils to obtain high resolution images of the carpal tunnel and median nerve. The imaging protocol consists of a fast T1-weighted gradient echo coronal localizer, an axial T1-weighted series and a short-tau inversion recovery (STIR) T2-weighted series. This protocol has established reliability and, because of the short imaging times, patients usually are in the MR scanner for only 15–20 minutes. Imaging parameters for the axial series are listed in table 2.

**Table 2:** Imaging Parameters

	T1- Weighted Axial	T2-Weighted Axial
Description	Spin Echo	STIR
flip angle	90	90
Echo train length		6
TE	Minimum/full	54
TR	450	3850
TI		160
Receiver bandwidth	16	12.8
Field of view	11	11
slice/skip	4/1	4/1
Saturation pulses	Superior/inferior	Superior/inferior
freq/phase matrix	256 × 256	256 × 224
freq direction	Right/left	Right/left
Number of excitations	2	2
Time	5:33	5:16
# images	20	11

The MRI is interpreted by a neuroradiologist without knowledge of the demographic data, clinical findings, or electrodiagnostic study findings. The key imaging variables are the degree and length of signal abnormality within the median nerve, flattening or swelling of the median nerve, although other qualitative and quantitative measurements will be made as well (table 3).

**Table 3:** Key MRI Imaging Variables

Median nerve
Signal (degree and length of signal abnormality)
Configuration (flattening or swelling)
Fascicular pattern
Flexor retinaculum bowing
Flexor tendon sheath interspaces

Patients receive a follow-up interview via telephone at 3, 6, 9 and 12 months. Hand specific symptom and functional disability scores, along with general health and psychological instruments are collected. For each interview, we attempt to contact participants a minimum of three times, varying the day and time of the call. In instances where we are unable to reach a person by phone, the survey instruments are mailed along with a postage paid return envelope. Some patients also consent to allow us to contact them for follow-up using e-mail.

All data are collected onto hardcopies and entered into an MS Access database using a web-based data entry system that requires double entry of data to reduce errors in transcribing. Error reporting to identify out of range answers, inconsistent replies and compliance monitoring is routinely performed.

We developed stopping rules using CTSAQ symptom scores, disability reporting, adverse event rates and rates of "red flag" answers to a question asking about thoughts of suicide on the SCL-90. A safety officer monitors these variables for group differences, and also monitors response variables, missing data, and protocol compliance.

A formal evaluation of efficacy will be conducted after 100 patients have been randomized to test for group differences in the CTSAQ symptom severity score. We adopted O'Brien-Fleming boundaries for discontinuation, and therefore maintain an overall type I error rate of 5%. Additional stopping criteria include a suicidal ideation rate difference of 10%, a difference in the change from baseline rate of functional disability (as measured by the CTSAQ function scale) of 20%, or a difference in the number of days lost from work of 20%. The nature and severity of the adverse events will also be considered individually by the safety officer. Finally, individual item response rates on all answered questionnaires with less than 20% being incomplete, failure to collect greater than 75% at three-month follow-up, or enrollment rates below the expected rate by more than 25% provided grounds for the safety officer to recommend changes or stop the study.

In the primary analysis the CTSAQ functional score at 12 months will be compared between the surgical and non-surgical treatment arms, using conventional t-tests and ANCOVA techniques to adjust for baseline values. The analysis will be conducted on an intention-to-treat basis.

Secondary analyses will include a comparison of secondary outcome measures (CTSAQ symptom severity, the SF-36 scales, time lost from work) for the two treatment arms. Adjusted analyses of CTSAQ functional status and secondary outcomes at 1 year will be conducted using linear regression methods. To characterize the time evolution in the primary and secondary outcomes we will use linear mixed models (or Generalized Estimating Equations in the case of categorical variables) to analyze the repeated measures obtained at all follow-up visits. Finally, we will use exploratory regression methods to determine if baseline disability, psychological factors, MRI variables, and electrodiagnostic measures correlate with clinical outcomes.

We will use aggregate results of patient outcomes on the CTSAQ and SF-36 scores to perform a descriptive analysis, uncontrolled for other baseline factors. We will explore factors that predict improvement in CTSAQ (symptom score and functional status) and general health scores as measured by the SF-36. Specific analysis and tests used will depend on the distribution of the tested values. We will also test the independent associations of the various mental health domains, the work-related risk factors, and physical findings to changes in the CTSAQ. Finally, we will use linear regression to test relevant associations while controlling for baseline demographics, co-morbidity and other important variables to identify factors that may predict improved outcomes at 1 year.

As important sub-analyses we will also determine the reliability of quantitative MR median nerve measurements, determine the correlation of symptoms and function in patients with these quantitative MR measurements as well as with electrodiagnostic studies (EDS), and construct a cost-effectiveness model to test the hypothesis that MRI is an efficient method for selecting patients with mild or moderate CTS who are likely to benefit from surgery.

The prospective cohort study by Katz et al. provided data on outcome differences between surgical and non-surgical patients [75]. They also used the CTSAQ as an outcome measure and found a 23–45% difference between surgical and non-surgical groups. Using pilot data we have calculated the mean reduction in CTSAQ function scores for 74 non-surgical patients as m0 = 0.264 (standard deviation = 0.670) and the mean reduction for 30 surgical patients as m1 = 0.818 (standard deviation = 1.033). The observed effect of surgery is m1 - m0 = 0.818-0.264 = 0.554 point greater reduction. We used the pilot data estimates of variance to calculate the sample size required to obtain sufficient power (80% or 90%) for various differences in means (m1-m0).

If we consider the difference in means observed for the pilot data (m1-m0 = 0.554) then 48+48 = 96 subjects are adequate for 80% power and a total of 64+64 = 128 are required for 90% power (refer to following table). However, since we are recruiting patients with less severe disease, we would expect smaller improvements in the scores from baseline to follow-up and hence, smaller effect sizes. Thus, it is reasonable to power the study to detect a difference in means of 0.4 rather than 0.5. Since our primary analysis will use the more efficient ANCOVA, these sample size estimates based on observed change scores (post-pre) are slightly conservative. This should allow for a small percentage of subjects lost to follow-up. Similar power calculations were conducted to assess the power to detect an impact on CTSAQ symptom scores. The pilot data suggest a 0.67 point greater reduction in symptoms for the surgical patients, and variance estimates lead to sample sizes of 30+30 = 60 total patients, and 40+40 = 80 total patients to detect 0.67 point difference (table 4). Our study will aim to enroll 100 patients into each arm of the study and this will be sufficient to detect the smallest clinically relevant differences.

**Table 4:** Power calculation In order to have 80% or 90% power for the analysis of reduction in function scores (change) we would need (per study arm)

	m1-m0			
	0.20	0.30	0.40	0.50
80% power	298	133	75	48
90% power	399	177	100	64

## Discussion

An RCT offers the best chance of answering, in an unbiased fashion, the relative efficacies of surgery compared with conservative therapy for patients with mild to moderate CTS. Our study is designed to test two main hypotheses: 1) that patients with mild or moderate CTS benefit from surgery more than conservative therapy, and; 2) that high-resolution wrist MRI accurately identifies which patients with mild to moderate symptoms are more likely to benefit from surgery. We expect our findings to be more generalizable to the primary care clinicians and include milder cases of CTS than Gerritsens' study. No previous study has sufficiently considered conservative treatment options of vitamin B6, ultrasound, anti-inflammatory drugs, and hand exercises in combination versus surgical treatment. Furthermore, our study offers the unique use of wrist MRI data to establish the diagnostic and predictive understanding of carpal tunnel syndrome.

## Pre-publication history

The pre-publication history for this paper can be accessed here:


